# Fatigue performance of endodontically treated premolars restored with direct and indirect cuspal coverage restorations utilizing fiber-reinforced cores

**DOI:** 10.1007/s00784-021-04319-3

**Published:** 2021-11-30

**Authors:** Márk Fráter, Tekla Sáry, Janka Molnár, Gábor Braunitzer, Lippo Lassila, Pekka K. Vallittu, Sufyan Garoushi

**Affiliations:** 1 Department of Operative and Esthetic Dentistry, Faculty of Dentistry, University of Szeged, Szeged, Hungary; 2 dicomLAB Dental Ltd., Szeged, Hungary; 3 Department of Biomaterials Science and Turku Clinical Biomaterials Center-TCBC, Institute of Dentistry, University of Turku, Turku, Finland; 4 City of Turku Welfare Division, Oral Health Care, Turku, Finland

**Keywords:** Premolars, Endodontically treated teeth, Short-fiber-reinforced composite, Fatigue survival, Indirect restoration, Fiber-reinforced post, Overlay

## Abstract

**Objectives:**

The aim of this in vitro study was to investigate the fatigue survival and fracture behavior of endodontically treated (ET) premolars restored with different types of post-core and cuspal coverage restorations.

**Materials and methods:**

MOD cavities were prepared on 108 extracted maxillary premolars. During the endodontic treatment, all teeth were instrumented with rotary files (ProTaper Universal) to the same apical enlargement (F2) and were obturated with a matched single cone obturation. After the endodontic procedure, the cavities were restored with different post-core and overlay restorations (*n* = 12/group). Three groups (A1–A3) were restored with either conventional composite core (PFC; control) or flowable short-fiber-reinforced composite (SFRC) core with/without custom-made fiber posts and without overlays. Six groups had similar post-core foundations as described above but with either direct PFC (B1–B3) or indirect CAD/CAM (C1–C3) overlays. Fatigue survival was tested for all restorations using a cyclic loading machine until fracture occurred or 50,000 cycles were completed. Kaplan-Meyer survival analysis was conducted, followed by pairwise post hoc comparisons.

**Results:**

None of the restored teeth survived all 50,000. Application of flowable SFRC as luting-core material with fiber post and CAD/CAD overlays (Group C3) showed superior performance regarding fatigue survival (*p* < 0.05) to all the other groups. Flowable SFRC with fiber post and direct overlay (Group B3) showed superior survival compared to all other direct techniques (*p* < 0.05), except for the same post-core foundation but without cuspal coverage (Group A3).

**Conclusions:**

Custom-made fiber post and SFRC as post luting core material with or without cuspal coverage performed well in terms of fatigue resistance and survival when used for the restoration of ET premolars.

**Clinical relevance:**

The fatigue survival of direct and indirect cuspal coverage restorations in ET MOD premolars is highly dependent on whether the core build-up is fiber-reinforced or not. The combination of short and long fibers in the form of individualized post-cores seems to offer a favorable solution in this situation.

## Introduction

Root canal treated (RCT) teeth are at an increased risk of failure-related fracture [1, 2]; therefore, the restorative material for their permanent restoration must be carefully chosen [3]. Dietschi et al. showed that the main reason for this increased risk is caries-related coronal destruction and the dentine loss that occurs during endodontic treatment [4]. These are quite significant in mesio-occluso-distal (MOD) cavities [5]. While a class I occlusal cavity causes only 5–20% loss of relative cuspal stiffness [6, 7], an MOD cavity causes an average of 63% loss of relative cuspal stiffness [8]. This is related mainly to the loss of both marginal ridges [9, 10]. As a result, fracture strength may be reduced by as much as 54% [11, 12]. Thus, intracoronal reinforcement in endodontically treated premolars with MOD cavities is essential to protect them against fracture [13, 14].

In the last 30 years, mainly fiber-reinforced composite (FRC) posts have been used to restore RCT premolars [15]. However, their actual reinforcing efficiency is still a matter of debate. Some studies managed to show increased fracture resistance when using FRC posts in endodontically treated premolar teeth [16–18], while others failed to do so [19, 20]. Yet other studies managed to show a positive effect only on failure patterns, but not on fracture resistance [21, 22].

The failure of conventional FRC posts to reinforce premolar teeth may have multiple reasons: their inadequate fit in the cervical region of the canal (leading to an increased amount of luting cement) [23, 24], poor bonding of the post material to the luting cement [25, 26], or even the biomechanically inadequate position of the fibers inside the canal [27]. Aiming to solve the above-mentioned shortcomings, the authors have proposed the Bioblock technique for the individualized restoration of irregular root canals [28–30]. In many cases, the Bioblock technique, utilizing either packable or flowable short fiber-reinforced composite (SFRC), has shown superior results compared to FRC posts [28, 29]. Furthermore, according to the latest research, those results may be further enhanced by using flowable SFRC as a luting material next to the FRC post inside the canal [31].

So far, cuspal coverage in the case of MOD cavities in root canal-treated teeth has been considered a mandatory safety measure by many [32–34]. However, with the development of individualized fiber-reinforced post and cores, the question arises whether cuspal coverage is necessary or not. It is also a yet unanswered question if there is a difference between direct (performed with particulate filled composite, PFC) and indirect cuspal coverage in this respect.

Thus, the purpose of this in vitro investigation was to evaluate whether a combination of an FRC post and flowable SFRC or flowable SFRC alone is ideal for the reinforcement of MOD endodontically treated premolars. In addition, the question of the effect of direct/indirect cuspal coverage on fatigue behavior was also investigated.

The null hypotheses were that (1) the tested individualized FRC post and core build-ups with and without cuspal coverage restorations would not differ from the control group in fatigue survival or (2) in their fracture pattern.

## Materials and methods

The study conformed to the tenets of the Declaration of Helsinki, and it was approved by the Regional Human Biomedical Research Ethics Committee at the University of Szeged, Hungary. All restorative materials used in this study are made by the same manufacturer and used according to the manufacturer’s instructions.

One hundred and eight maxillary premolar teeth, extracted for periodontal or orthodontic reasons, were selected for this investigation. The newly extracted premolars were directly inserted in sodium hypochlorite (5.25%) for 5 min and then kept in saline (0.9%) for a maximum of 12 weeks at room temperature before use. After extraction, with the aid of hand scalers, the root surface was cleaned from the covered soft tissue. The teeth selection criteria regarding their integrity and radicular dimensions were the same as in our previous method [31]. Regarding coronal dimensions, 90% of the teeth ranged between 9 and 10 mm bucco-palatally, measured at the widest bucco-palatal dimension. The average mesio-distal dimension was between 7 and 7.5 for 90% of the samples. Ten percent maximum deviation was allowed in the remaining 10% of the samples. The height of the crowns was between 7.5 and 8 mm in 90% of the samples, and + /0.5 mm deviation was allowed in the rest of the samples.

The teeth were randomly distributed across 9 study groups of 12 specimens each (A1 = control, A2–3, B1–3, C1–3). Specimens with average dimensions (within the above-mentioned 90%) were randomly distributed across the 9 groups, and specimens with non-average dimensions (the remaining 10%) were evenly distributed across the same 9 groups. MOD cavity preparation and root canal treatment were performed by the same trained operator. A standardized MOD cavity was prepared using a round-end parallel diamond (881.31.014 FG, Brasseler USA Dental, Savannah, GA) with water cooling. So the cavity was prepared so that the buccopalatal width of the occlusal isthmus was one-third of the intercuspal width, and the proximal box width was half of the buccopalatal width of the crown. The gingival floor was located 1 mm above the cemento-enamel junction (CEJ). All internal angles were rounded and the cavosurface margins were at 90°. After finalizing the MOD cavity preparation, access cavity preparation was carried out with a round-end diamond bur (850–014 M SSWhite, Lakewood, NJ, USA) with water cooling, and root canal treatment was performed in the prepared teeth.

The working length was defined utilizing the direct method, that is, by subtracting 1 mm from the real root length, which was determined by introducing a number 10 K-file (Maillefer-Dentsply, Ballaigues, Switzerland) until it was visible through the apical foramen. The root canals were prepared using rotary ProTaper Universal files (Maillefer-Dentsply). The ProTaper sequence (S1, S2, F1, F2) was used for the preparation of the working length. Irrigation was done after every instrument with 2 ml of 2.5% NaOCl solution, and the canal space was filled with irrigant during the instrumentation phase. After root canal cleaning and shaping, the roots were dried using 96% alcohol and paper points. Root canal obturation was done by matched single-cone obturation with a master cone (F2 gutta-percha, Maillefer-Dentsply) and sealer (AH plus; Dentsply De Trey GmbH, Konstanz, Germany). The gutta-percha was cut back to the level of the orifice, and the access cavity was temporarily filled with Fuji Triage Pink (GC Europe, Leuven, Belgium). Fuji Triage Pink was applied to the apical part of the root to prevent leakage through the apex. The teeth were stored wet in an incubator (mco-18aic, Sanyo, Japan) for 1 week (at 37 °C, 100% relative humidity). After this, the temporary material was removed, and the MOD cavity including the access cavity was refreshened with a diamond bur.

In Groups A2-3, B2-3, and C2-3, post space preparation was carried out by a 1.2 GC Fiber Post drill to a depth of 6-mm apical from the root canal orifice, as proposed in one of our previous studies [31]. After cutting back the gutta-percha, the root canal was washed with chlorhexidine and dried with paper points.

Finally, in all groups marked “B” and “C,” all cusps were reduced by 2 mm of their original height.

All specimens received the same adhesive treatment. A Tofflemire (1101C 0.035, KerrHawe, Bioggio, Switzerland) matrix band was applied before the adhesive treatment of the cavity and the root canal, and the enamel was selectively acid-etched with 37% phosphoric acid for 15 s and washed with water. The coronal cavity and the root canal were rinsed with 2 ml of water and dried with paper points and air. A dual-cure one-step self-etch adhesive system (G-Premio Bond and DCA, GC Europe) was used for bonding, according to the manufacturer’s instructions, using a microbrush-X disposable applicator (Pentron Clinical Technologies, LLC, USA). Excess adhesive was eliminated by suction drying (Evacuation Tip–Starryshine, Anaheim, CA, USA) applied approximately 0.5 cm from the occlusal cavity (without contact). Excess adhesive resin at the bottom of the canal was eliminated using a paper point. The adhesive was light-cured for 60 s using an Optilux 501 quartz-tungsten-halogen light-curing unit (Kerr Corp., Orange, CA, USA). The light-curing tip was always in close contact (1–2 mm) with the tooth surface. The average power density of the light source, measured with a digital radiometer (Jetlite light tester, J. Morita USA Inc. Irvine, CA, USA) before the bonding procedure, was 840 ± 26.8 mW/cm^2^.

In the control group and group B1, the missing interproximal walls were built up with conventional composite (G-aenial Universal Injectable, shade A3, GC Europe), while in groups A2, A3, B2, and B3, the missing interproximal walls were built up with flowable SFRC (everX Flow, dentine shade, GC Europe) using the centripetal technique, thus transforming the MOD cavity into a class I cavity. This interproximal wall was light-cured for 40 s.

**Fig. 1:**
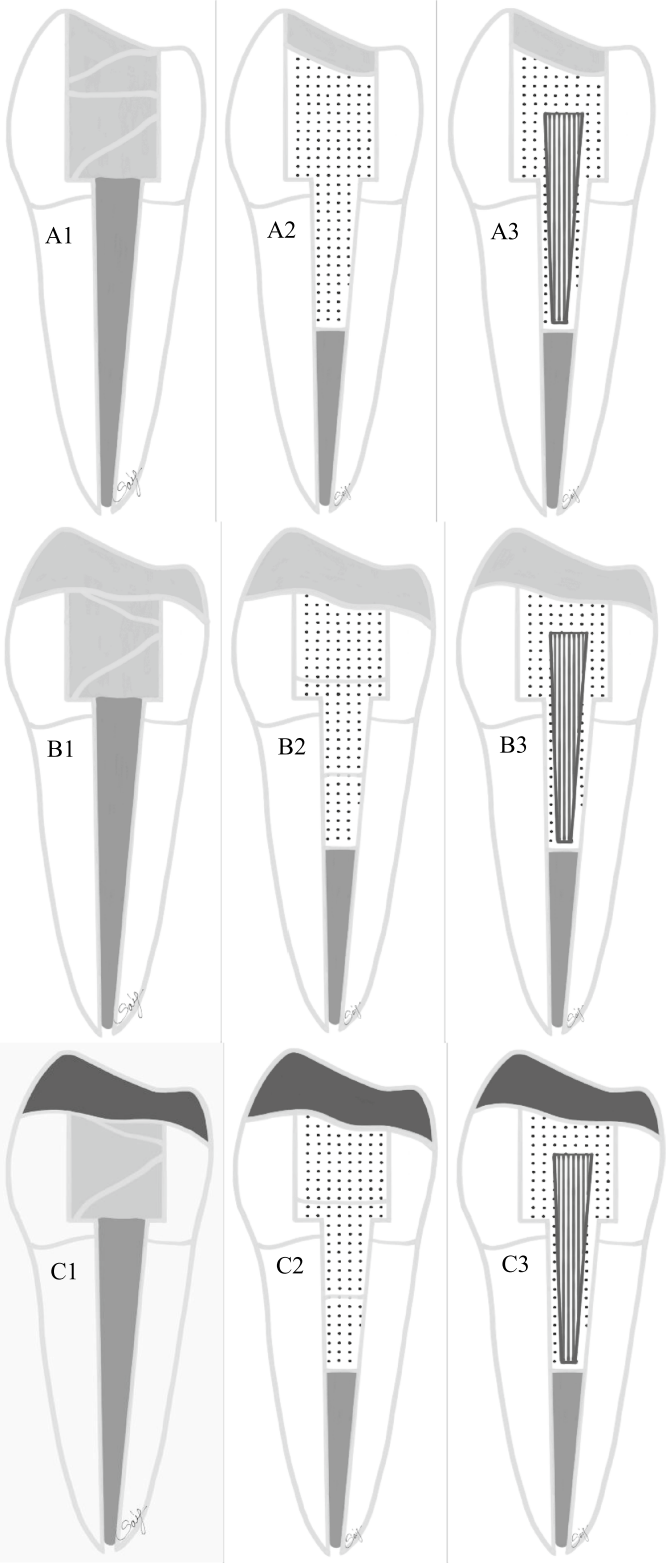
Diagram showing the test groups (A1–C3) restored with different approaches with various post-core and overlay restorations. A1 (control), only PFC without overlay; A2, SFRC + PFC; A3, post + SFRC + PFC; B1, PFC + direct overlay; B2, SFRC + direct overlay; B3, post + SFRC + direct overlay; C1, PFC + indirect overlay; C2, SFRC + indirect overlay; C3, post + SFRC + indirect overlay

The teeth/groups were restored according to different restorative approaches (see Fig. 1 and Table 1).

**Table 1:** Different post-core and cuspal coverage restorations (*n* = 12/group)

Group	Post-core	Cuspal coverage
A1 (control)	PFC core	No
A2	SFRC	No
A3	Post + SFRC	No
B1	PFC core	Direct PFC
B2	SFRC	Direct PFC
B3	Post + SFRC	Direct PFC
C1	PFC core	CAD/CAM
C2	SFRC	CAD/CAM
C3	Post + SFRC	CAD/CAM

### Group A1 = control group

The cavities were restored with conventional PFC composite material (G-aenial Universal Injectable) applied with an oblique incremental technique. The material was placed in consecutive 2-mm-thick increments. Each increment was light-cured from the occlusal surface for 40 s.

### Group A2

The cavities and the 6-mm-deep post space were reconstructed with the Bioblock technique described by Fráter et al. [28], building a direct layered post and core from flowable SFRC. The thickness of the increments was approximately 4 mm, and a microbrush-X disposable applicator (Pentron Clinical Technologies, LLC, USA) was used. A light-transmitting FRC post (1.2 mm GC Fiber post, GC Europe) was inserted into the post space to aid the transmission of the light to the apically positioned layers. The “light transmitting” post was withdrawn 0.5–1 mm from the surface of the uncured SFRC layer not to have direct contact with it. This apical layer was light-cured through the fiber post for 80 s. The rest of the cavity was restored with two 4-mm-thick layers of flowable SFRC (everX Flow, bulk shade, GC Europe). The material was placed to a level according to the anatomy of the dentine, leaving 2 mm occlusally for the final PFC composite. These SFRC increments were light-cured from the occlusal surface for 40 s. The last occlusal layer was conventional composite material (G-aenial Universal Injectable) covering the SFRC as described in Group A1.

### Group A3

The teeth received a custom-made unidirectional FRC post (everSTICK POST, GC Europe). Before the adhesive treatment, the posts of 1.2-mm diameter were tried in and cut to a length of 2 mm below the level of the occlusal cavity margins with sterile scissors. Luting of the posts and the core build-up was performed with flowable SFRC as described by Fráter et al. [31]. Flowable SFRC was applied in approx. 4-mm-thick layers into the post space. After insertion of the post, light-curing was performed for 60 s. The coronal portion of the cavity was restored as described in Group A2.

### Group B1

The cavities were restored with conventional PFC composite material (G-aenial Universal Injectable) as described in Group A1 to the level of the occlusal reduction. The previously reduced cusps were built back by conventional PFC composite with the aid of a silicon index.

### Group B2

The cavities and the 6-mm-deep post space were reconstructed with flowable SFRC as in Group A2 to the level of reduction. The previously reduced cusps were built back by conventional PFC composite with the aid of a silicon index.

### Group B3

The cavities and the 6-mm-deep post space were reconstructed with a custom-made FRC post (everSTICK POST) as in Group A3 to the level of reduction. The previously reduced cusps were built back with the conventional composite material with the aid of a silicon index.

### Group C1

The cavities were restored with conventional composite material as described in Group A1 (G-aenial Universal Injectable) in the form of a core build-up, leaving 2-mm space for the overlay on the occlusal and also on the interproximal surfaces.

### Group C2

The cavities and the 6-mm-deep post space were reconstructed with flowable SFRC as in Group A2 to the level of reduction in the form of a core build-up, leaving 2-mm space for the overlay on the occlusal and also on the interproximal surfaces.

### Group C3

The cavities and the 6-mm-deep post space were reconstructed with a custom-made FRC post (everSTICK POST) as in Group A3 in the form of a core build-up, leaving 2-mm space for the overlay on the occlusal and also on the interproximal surfaces.

Groups C1, C2, and C3 then received indirect CAD/CAM overlays according to the following steps:

After refining the cavity margins, a polyether impression (Permadyne, 3 M ESPE) was taken of each prepared specimen, using a simultaneous mixing technique according to the manufacturer’s instructions. The impressions were poured with type IV dental stone (FUJIROCK, GC Europe) and 2-mm-thick CAD/CAM composite resin overlays (CERASMART 270, GC Europe) were prepared by the same technician for each prepared specimen.

Luting was performed as follows: the intaglio surface of the composite overlays was treated with hydrofluoric acid for 20 s. After rinsing and drying, the overlays were silanized (G-Multi PRIMER, GC Europe) and dried. As for the teeth, the enamel margins were etched with 37% phosphoric acid for 30 s, rinsed with water, and air-dried. The surface of the composite core was roughened with a diamond bur, and a bonding agent was applied on the core (G-Premio Bond). Then the same bonding agent was applied on the intaglio of the restoration and left undisturbed for 10 s. Using an air syringe, the surface of the restoration was dried for 5 s with maximum air pressure. The overlays were luted with dual-cure resin cement (G-CEM LinkForce, GC Europe). The luting agent was applied onto the intaglio surface of the overlays, and the overlays were applied on the teeth under finger pressure until complete adaptation. After removing the excess material, glycerine gel (DeOx Gel, Ultradent Products Inc., Orange, CA, USA) was applied, and photopolymerization from each side for 40 s with Optilux 501 was performed.

Embedding and the simulation of the periodontal ligaments were carried out as described in our previous studies [31, 35]. During the mechanical testing, the specimens were submitted to an accelerated fatigue-testing protocol [29, 31] by a hydraulic testing machine (Instron ElektroPlus E3000, Norwood, MA,USA) at an angle of 135 degrees to the long axis of each tooth. Cyclic isometric loading was applied on the triangular ridge of the buccal cusp of the tooth using a round-shaped metallic tip (with a diameter of 5 mm). To aid the proper positioning of the testing tip, the palatal cusp was slightly reduced. The cyclic load was applied at a frequency of 5 Hz, starting with gradually increasing static loading till 100 N in 5 s, followed by cyclic loading in 100 N steps, up to 1000 N, 5000 cycles per step. The specimens were loaded until fracture occurred or 50,000 cycles were reached. The total number of survived cycles was recorded for each specimen for the survival analyses.

After recording failure load, each specimen was visually examined for the type and location of the failure, as well as the direction of failure. According to Scotti and co-workers, a distinction was made between restorable and non-restorable fractures under an optical microscope (Carl Zeiss Omni Pico, Oberkochen, Germany) with a two-examiner agreement. A restorable fracture is above the CEJ, meaning that in case of fracture, the tooth can be restored, while a non-restorable fracture extends below the CEJ, and the tooth is likely to be extracted [36].

Statistical analysis was performed in SPSS 23.0 (IBM Corp., Somers, NY, USA). Kaplan-Meyer survival analysis was conducted, followed by pairwise post hoc comparisons (Breslow). The frequency of restorable and non-restorable fractures was calculated for each group.

## Results

**Fig. 2:**
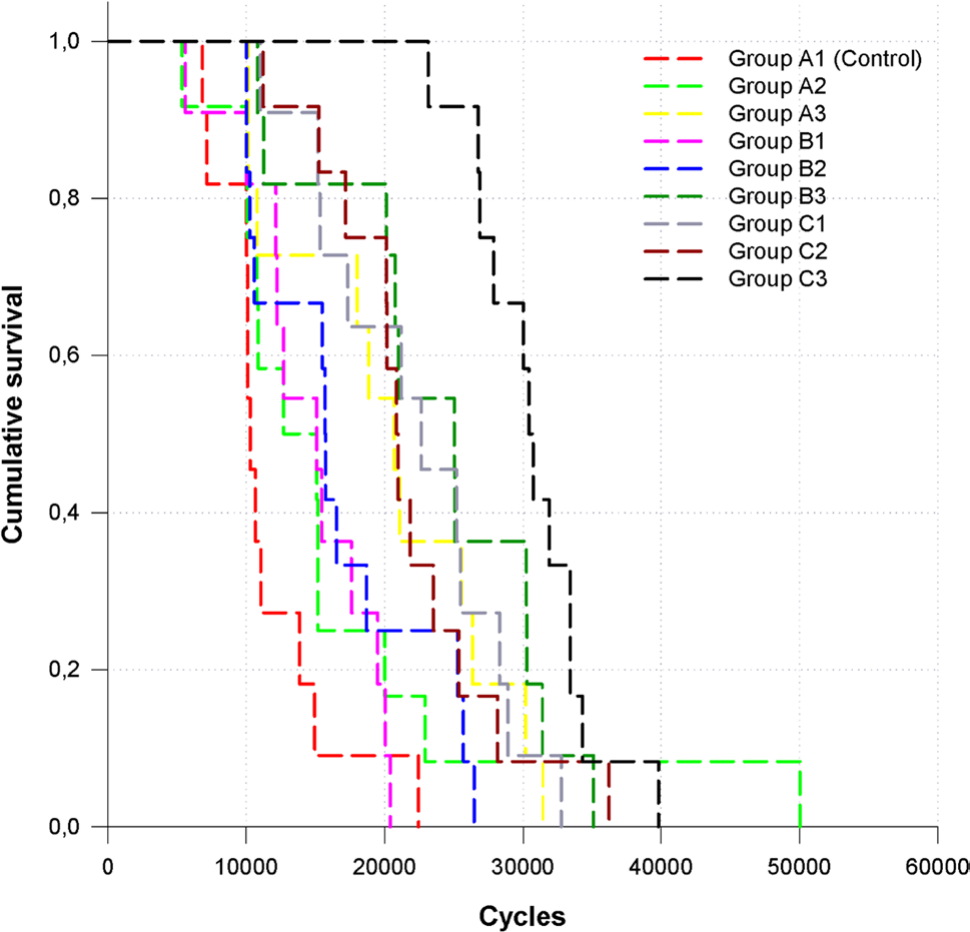
Fatigue resistance survival curves (Kaplan–Meier survival estimator) for all tested groups

The Kaplan–Meier survival curves are presented in Fig. 2. Table 2 shows the descriptive characterization of the survival as the mean and median number of survived cycles for each tested group. During the mechanical fatigue testing, the embedding surrounding the teeth broke in 5 specimens, but the teeth did not; thus, those teeth were excluded from the study. Table 3 shows the *p*-values for the group-wise comparisons. Group C3 presented the highest survival rate, while the control group presented the lowest survival rate in this study. Furthermore, Group C3 showed significantly higher survival values compared to all other tested groups (*p* < 0.05). The control group did not differ statistically from Groups B1 and A2 (*p* = 0.076 and *p* = 0.135, respectively). The rest of the tested groups showed statistically significantly higher survival rates compared to the control group (*p* < 0.05). Group B3 showed higher survival rates than the control group, and Groups A2, B1, and B2 (*p* < 0.05), however, did not differ from Groups A3, C1, and C2 in terms of survival.

**Table 2:** Average of survived load cycles and their standard deviations

Group	Mean	*N*	Std. Deviation
Group A1 (control)	11,590.55	11	4316.577
Group A2	16,527.92	12	11,556.415
Group A3	20,296.00	11	7683.110
Group B1	14,621.82	11	4628.846
Group B2	16,699.75	12	6214.033
Group B3	23,735.55	11	7933.014
Group C1	22,124.82	11	6782.084
Group C2	21,739.92	12	6380.115
Group C3	30,728.50	12	4358.524
Total	19,848.63	103	8644.093

**Table 3:** *p* values of pairwise log-rank post-hoc comparisons among tested groups (Kaplan–Meier survival estimator followed by log-rank test for cycles until failure or the end of the fatigue loading)

GroupREC	Group A1 (control)		Group A2		Group A3		Group B1		Group B2		Group B3		Group C1		Group C2		Group C3	
	Chi-square	Sig	Chi-square	Sig	Chi-square	Sig	Chi-square	Sig	Chi-square	Sig	Chi-square	Sig	Chi-square	Sig	Chi-square	Sig	Chi-square	Sig
Group A1 (control)			2.235	.135	7.647	.006	3.142	.076	4.084	.043	12.672	.000	13.956	.000	14.402	.000	22.000	.000
Group A2	2.235	.135			2.580	.108	.093	.760	,653	.419	6.048	.014	6.724	.010	7.337	.007	14.371	.000
Group A3	7.647	.006	2.580	.108			3.384	.066	1.828	.176	1.317	.251	.308	.579	.136	.713	11.951	.001
Group B1	3.142	.076	.093	.760	3.384	.066			.305	.581	8.270	.004	6.488	.011	9.004	.003	22.000	.000
Group B2	4.084	.043	.653	.419	1.828	.176	.305	.581			4.762	.029	2.769	.096	3.525	.060	19.714	.000
Group B3	12.672	.000	6.048	.014	1.317	.251	8.270	.004	4.762	.029			.129	.720	.634	.426	5.263	.022
Group C1	13.956	.000	6.724	.010	.308	.579	6.488	.011	2.769	.096	.129	.720			.183	.669	9.899	.002
Group C2	14.402	.000	7.337	.007	.136	.713	9.004	.003	3.525	.060	.634	.426	.183	.669			11.491	.001
Group C3	22.000	.000	14.371	.000	11.951	.001	22.000	.000	19.714	.000	5.263	.022	9.899	.002	11.491	.001		

**Fig. 3:**
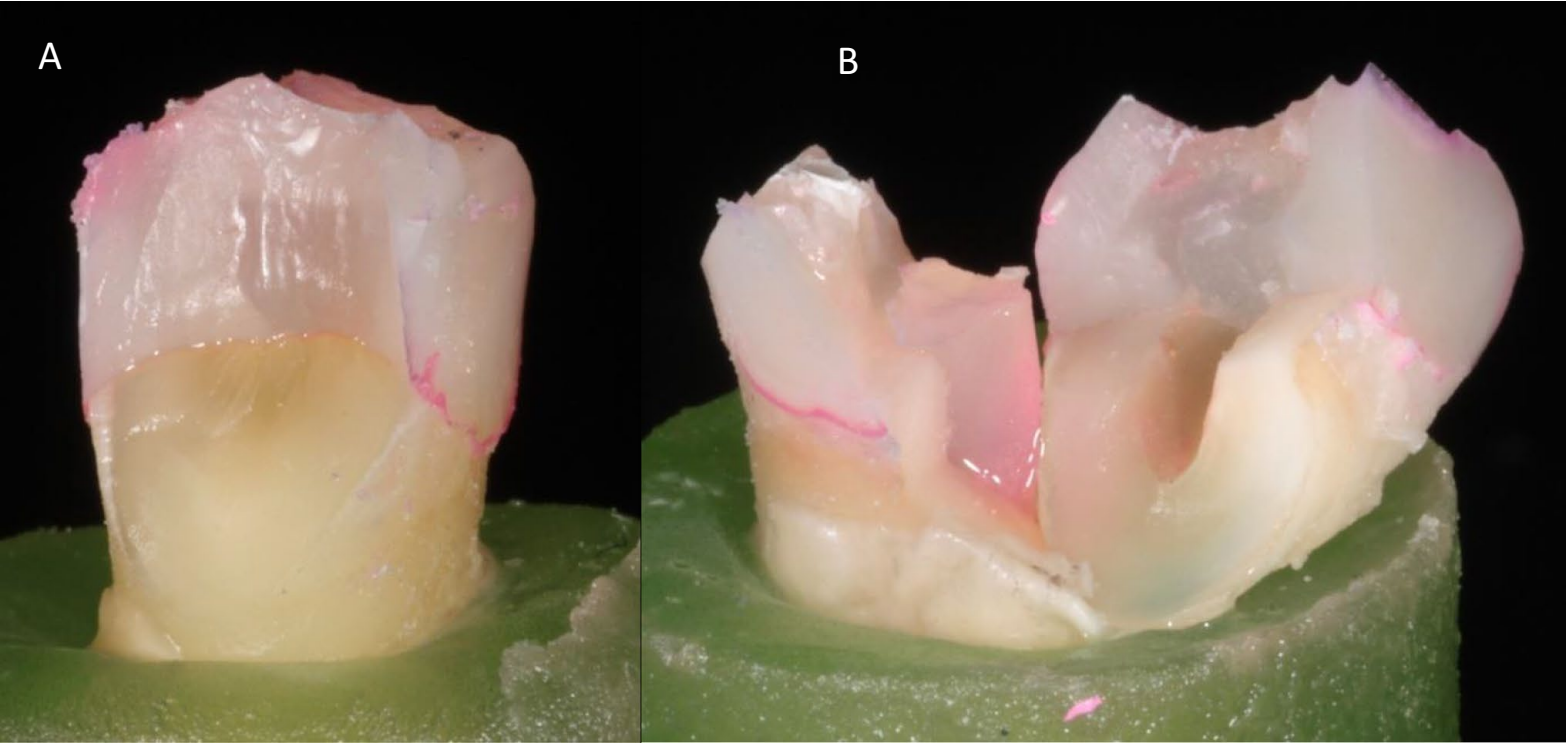
Photographs of restorable (**A**) and non-restorable (**B**) fracture mode of the tested specimens

Regarding the type of fractures, all restored groups showed dominantly non-restorable fractures (Table 4, Fig. 3). Non-restorable fractures ended either (a) below or at the bone level emerging from the buccal or palatal wall or (b) spreading into the root splitting the tooth. Inter-rater agreement regarding fracture patterns was assessed by Cohen’s κ. As there was an extremely low number of cases (*N* = 3) where the raters did not agree, and this meant that in most of the groups the agreement was 100%, calculated kappa was calculated for the entire sample. The test indicated almost perfect agreement (κ = 0.93). The highest number of non-restorable fractures was observed in Group C3.

**Table 4:** The distribution of fracture mode among the tested groups

		Group A1 (control)	Group A2	Group A3	Group B1	Group B2	Group B3	Group C1	Group C2	Group C3
Non-restorable	Count	9	8	10	9	9	6	8	7	11
	% within group	81.8%	66.7%	90.9%	81.8%	75.0%	54.5%	72.7%	58.3%	91.7%
Restorable	Count	2	4	1	2	3	5	3	5	1
	% within group	18.2%	33.3%	9.1%	18.2%	25.0%	45.5%	27.3%	41.7%	8.3%

## Discussion

This study focused on the effect of individualized fiber-reinforced designs with and without cuspal coverage on fatigue survival. The question of whether direct cuspal coverage can be as effective as its indirect counterpart was also addressed.

In our study, cyclic loading was used instead of static load-to-fracture testing. When testing tooth-restoration units, cycling loading, by generating repetitive forces just as during normal chewing, corresponds better to the real clinical situation. Also, as stated by Le Bell-Rönnlöf, fatigue more often leads to root fracture compared to static forces [27]. Accelerated fatigue testing has been used in many studies [37–43]. Accelerated fatigue was introduced as a rational middle ground between the load-to-fracture test and the more sophisticated and time-consuming fatigue tests [44]. In this study, maxillary RCT premolars with MOD cavities were used as they present unfavorable anatomy in crown volume and crown-to-root proportion, making them more susceptible to cusp fractures compared to other posterior teeth when exposed to occlusal load [16, 45]. Furthermore, during mastication, premolars are subjected to a more detrimental combination of lateral forces, namely, a combination of shear and compressive forces, which increases the risk of future cusp fracture [46]. In our study, the tested specimens received oblique loading, which appears to be the worst-case scenario in terms of the loading direction in mechanical testing of RCT teeth as described by Wandscher et al. [47].

In the past years, the Bioblock technique utilizing packable or flowable SFRC showed remarkable results in restoring root canal-treated teeth in our studies [28, 29]. However, when short and long fibers are combined for individualized reinforcement, so far superior results can be seen [31, 48, 49].

Choosing the best material for core build-up in root canal-treated teeth is a hot, yet unsolved topic. In a current systematic review and meta-analysis, Zarow et al. concluded that highly filled core composite materials could be a better option compared to classic composite materials [50].

In contrary to this, Stavridakis et al. recommend the usage of flowable composites as a core build-up material next to an FRC post; however, they made this recommendation in connection with anterior cases without a ferrule [51]. Fiber-reinforced composites outperformed non-fiber-reinforced composites in many cavity formations and situations. Furthermore, since flowable SFRC has shown superior mechanical features compared to packable SFRC [52], it seems logical to use the flowable variant also as a core build-up either alone or next to an FRC post. In the study of Lassila et al. and Uctasli et al., flowable SFRC yielded promising results when used as a core build-up together with an FRC post [48, 53]. More importantly from a clinical point of view, the handling and application of the flowable SFRC are much more convenient for a core build-up either alone or together with an FRC post compared to packable composites, be they fiber-reinforced or not.

In our study, the control group (composite filling) was characterized by the lowest and group C3 (indirect overlay placed on a core containing both short and long fibers) with the highest survival rates. When looking at the direct restorations without cuspal coverage in this study, Group A3 (flowable SFRC with an individually made FRC post) showed superior survival compared to the control group (*p* = 0.006). Thus, the first null hypothesis was rejected. This is in line with our previous findings [31] in RCT premolars with mesio-occlusal (MO) cavities. In this study, the Bioblock technique without cuspal coverage (Group A2) was not significantly superior to the non-fiber-reinforced composite filling (control group). This is apparently contrary to the findings of Shah et al. [54] and Eapen et al. [45]. However, they used SFRC only in the coronal cavity and performed load-to-fracture testing. Garlapati and colleagues also found SFRC to yield significantly higher fracture resistance values in RCT MOD cavities compared to PFC composite filling [55]. However, they used a load-to-fracture test, and the tested specimens were molar teeth, which could explain the difference.

As for the optimal treatment of RCT MOD cavities, the literature seems to agree that indirect cuspal coverage is indicated for safety reasons [32–34]. However, with the advancement of adhesive techniques and composite materials, the possibility of direct cuspal coverage with PFC has arisen. This technique eliminates the need for a dental technician, making the restorative procedure less time-consuming and less expensive. Direct cuspal coverage as a successful restorative option has been documented in clinical settings [56–58]. When considering the results on direct cuspal coverage, direct cuspal coverage on PFC composite filling (Group B1) did not prove to be significantly more beneficial than the methods without cuspal coverage (Control, Groups A2-3). This is in agreement with the results of Mohammadi et al. [2] but contradicts the findings of Fennis et al. [59] and ElAyouti et al. [60]. Interestingly, when direct cuspal coverage was combined with the Bioblock technique (Group B2), the survival significantly improved as compared to the control group (*p* = 0.043). It seems obvious that this can be put down to the strengthening effect of incorporating flowable SFRC into the restoration and providing cuspal coverage simultaneously. It is noteworthy that not only did the Bioblock technique with direct cuspal coverage (Group B2) not differ from the custom-made FRC post with flowable SFRC (Group A3) in terms of survival, but it was not significantly different from the indirect cuspal coverage restorations on either composite (Group C1) or Bioblock core build-up (Group C2) either. These results could suggest that the Bioblock technique combined with direct cuspal coverage or a custom-made FRC post with flowable SFRC without cuspal coverage could be alternatives to indirect cuspal coverage. To our knowledge, this is a novel finding under in vitro conditions. These FRC-based approaches can provide a safe direct restorative solution for patients with RCT MOD cavities in the premolar area. While there was no statistically significant difference between the Bioblock technique and the custom-made FRC post with flowable SFRC when not using cuspal coverage (Groups A2 and A3), a significant difference was found when direct cuspal coverage was applied with the same two FRC-based approaches (Groups B2 and B3, *p* = 0.029). It seems that the use of long fibers in the form of a custom-made FRC post (everSTICK POST) is beneficial in premolar RCT teeth. This is in line with our previous findings [28, 31]. The explanation is probably that long fibers provide additional protection against the compressive, shear, and even tensile forces that premolars must withstand due to their position in the arch. Thus, it seems that in regions where teeth are potentially exposed to high shear and tensile forces (typically the anterior and premolar regions), it is recommendable to include long fibers in the restoration of RCT teeth [49]. However, these recommendations should be verified under in vivo conditions in the future.

It is especially important to note that while the restored teeth are in function, damage-causing tensile stress occurs especially on their outer surface, so the tougher material(s) should be placed there rather than in the middle of the root canal where the neutral axis of stress is located. This approach, i.e., the individual fiber post concept, has been used successfully with continuous long glass fibers [61]. Short fiber-reinforced core composites, on the other hand, showed the ability to re-direct and stop crack propagation within the materials [31]. The presence of such energy-absorbing and stress-distributing fibers allows crack propagation to be deflected away from the bulk of the material and toward the peripheries [62–64].

In this study, individualized fiber-reinforced post and cores were placed 6-mm deep into the root canal, as proposed in our most recent study [31]. So far many studies have pointed out that the length of FRC posts inside the canal is not a critical factor [65–68], in contrast to the situation when metal posts are used. According to Meyenberg, it could be enough for a clinically satisfactory outcome to have approximately 7 mm of FRC post inserted inside the canal with approximately 4 mm of the same post providing the coronal retention to the core build-up [69].

This could even be more relevant in the case of individualized FRC post and cores as they fit better in the critical cervical area in the root canal (since the amount of fibers are maximized while the luting cement is minimized). Subsequently, the increased amount of fibers can potentially compensate for the reduced post insertion depth in these cases. It is to be pointed out that the 6-mm post depth in this study was chosen on purpose, to allow direct comparison with the results of our previous study on restoring ET premolar teeth. The authors consider this a strength of this study.

Although there is evidence on the concept of using shorter (up to 7 mm) individualized in the cervical part FRC posts, it has to be very clear that we are talking about single tooth restoration receiving inlays/onlays or crowns. The efficacy of this type of post and core to be used in endodontically treated abutment teeth of fixed partial dentures, especially the long span or in abutments of removable partial dentures, is not supported by the current data and warrants further investigation.

Direct cuspal coverage did not improve the fatigue survival of specimens restored with the custom-made FRC post with flowable SFRC (Group B3 compared to A3). This could be attributed to the unique properties that emerge as the custom-made RFC post and the flowable SFRC are combined. The custom-made FRC post is made of unidirectional fibers (E-glass) impregnated with a combination of bisphenol A-glycidyl methacrylate as the cross-linked phase and polymethyl methacrylate as a linear phase, forming together a semi-interpenetrating polymer network (semi-IPN) [70]. Since this semi-IPN structure contains both cross-linked and linear polymer phases, it ensures a stable adhesion to resin materials used for luting. In Groups A3, B3, and C3, flowable SFRC was used for post luting. This is an important aspect as this way either long (FRC post) or short fibers (flowable SFRC) will be in direct contact with the wall of the root canal, where the detrimental stress occurs upon loading [27, 71]. According to our previous studies, SFRC materials inside the root canal, including flowable SFRC used for luting next to an individual FRC post, can be safely light-cured [28, 29, 31]. This could be traced back to multiple reasons, namely, the light transmission of the FRC post [72], the transparency of the SFRC materials, and the scattering of light by the short fibers [73]. As a result, SFRC materials can be safely light-cured to a depth of approximately 5 mm even in cavities and without the aid of an FRC post [74–76].

The highest survival rate was seen when indirect cuspal coverage was provided with the individual FRC post and flowable SFRC combination (Group C3). More importantly, this restorative method led to significantly better survival rates compared to all the other approaches. Within our study setup, this was the only case when a significant difference was found between direct and indirect cuspal coverage restorations utilizing fiber-reinforced core build-ups.

Regarding the fracture patterns, all groups were characterized by predominantly non-restorable fractures. Therefore, the second null hypothesis was accepted. It is clearly shown in the literature that the brittleness of the conventional PFC materials generates bulk fracture propagating easily through the whole thickness of the restoration, reaching the tooth substance to be propagated further [62, 63]. Thus, the basic characteristics of the PFC materials do not offer significant protection against fatigue crack propagation. In contrast, the SFRC core supports the PFC layer and acts as a crack-stopper [35, 62, 63]. To be able to reinforce PFC, the integral toughness of the SFRC-core should be superior to that of the PFC surface layer [77]. If the SFRC-core is considered primarily a crack-stopper, it is the distance between the stress initiation point on the surface and the SFRC-core that matters. Thus, the top surface PFC thickness might contribute to the fatigue survival and failure mode. In this study, a 2-mm thick PFC surface layer was utilized, and this might explain the non-restorable fracture pattern even in the SFRC-core groups. This is in line with the results of previous investigations which showed the importance of SFRC and PFC layer thickness [63, 77, 78].

To maximize the amount of fibers and toughness, flowable SFRC (dentine shade) was used to build up the missing interproximal walls in Groups A2, A3, B2, and B3. It is to be noted that SFRC is supposed to be used as a bulk base or core foundation and should not be used as final surface restoration, as per the manufacturer’s instructions. At the same time, several laboratory investigations and clinical reports showed that the filler loading of these materials did not affect either the wear or the gloss of the composite restorations [52, 79–82].

This was an in vitro study carried out on extracted human teeth. While the study has several strengths, it also has its limitations. For instance, the dynamic loading tests were not carried out in a fluid chamber, which would have modeled the intraoral environment more closely. The authors are aware of this limitation, and the reader is advised to interpret the results with this in mind. Furthermore, our results need to be verified in vivo, and other aspects should also be examined, such as wear, color stability, and the quality of interproximal contacts in time. Finally, ceramic overlays should also be tested on the proposed individualized fiber-reinforced post and core designs.

## Conclusions

Custom-made fiber post and SFRC as post luting core material with or without cuspal coverage performed well in terms of fatigue resistance and survival when used for the restoration of ET premolars.

The performance of indirect CAD/CAM overlay restorations was superior to direct composite overlay restorations.
